# DSA-DET: a tea disease detection algorithm based on dynamic spatial pyramid and polarized linear attention

**DOI:** 10.3389/fpls.2026.1818867

**Published:** 2026-05-18

**Authors:** Haoxiang Dai, Jiaxin Lv, Chenlu Sun, Guotao Wang, Xin Guan, Ziheng Yang

**Affiliations:** 1 Electrical Engineering College, Heilongjiang University, Harbin, China; 2 School of Computer Science and Artificial Intelligence, Zhengzhou University, Zhengzhou, China; 3 Data Science and Technology College, Heilongjiang University, Harbin, China

**Keywords:** deep learning, multi-scale feature fusion, RT-DETR, smart agriculture, tea disease detection

## Abstract

**Introduction:**

Traditional tea disease detection methods suffer from low efficiency and strong subjectivity, while existing deep learning approaches often demonstrate inadequate detection accuracy and poor real-time performance in complex and variable environments.

**Methods:**

Here, we present an intelligent tea disease detection method based on an improved Real-Time Detection Transformer, named DSA-DET. We propose a backbone network based on dynamic attention spatial pyramid modeling to achieve more accurate collaborative modeling of local features and global context. We design an encoder combining polarized linear attention with parallel spatial enhancement networks and multi-scale adaptive enhancement to improve feature extraction capabilities. We further develop an upsampling module employing efficient spatial-channel upsampling and shift mixing mechanisms to enhance the quality of reconstructed features.

**Results:**

Experimental results show that the improved model achieves a precision of 94.73%, a recall of 89.65%, and an mAP^50^ of 93.68%. Compared to the baseline model RT-DETR-R18, its precision is improved by 3.56%, recall by 2.96%, and mAP^50^ by 3.02%; meanwhile, the model maintains a lightweight parameter scale of 15.4M and a real-time detection speed of 71.5 FPS.

**Discussion:**

The improvement scheme in this study successfully enhances detection accuracy while maintaining a good balance between model complexity and inference speed, providing a practical and reliable technical solution for the intelligent diagnosis of tea diseases.

## 1 Introduction

As one of the most popular beverages worldwide, tea not only possesses health benefits such as enhancing immunity and preventing various diseases (Fang et al., 2019), but also serves as a vital cash crop in numerous tea-producing countries and regions (Bag et al., 2022; Xia et al., 2020; Bora et al., 2019), playing a significant role in promoting rural economic development and increasing farmers’ income (Wei et al., 2024; Xu et al., 2021). However, tea plants are highly susceptible to various diseases during their growth, including tea blister blight, tea brown blight, tea gray blight, and tea red rust. These diseases can cause approximately a 20% annual loss in tea production, posing a severe threat to the sustainable development of the tea industry (Liu et al., 2020; Hu et al., 2019). Conventional disease detection primarily relies on manual visual inspection, which is not only time-consuming and labor-intensive with low efficiency, but also heavily dependent on the professional experience of inspectors, resulting in considerable subjectivity and high misdiagnosis rates (Bao et al., 2022). Therefore, developing efficient, accurate, and real-time intelligent detection technology for tea diseases holds significant theoretical value and practical implications for achieving early disease warning, precision pesticide application, and smart tea plantation management. Traditional tea disease detection methods are predominantly based on image processing and machine learning techniques. Barbedo et al (Barbedo et al., 2016) proposed a plant disease recognition method utilizing color transformation, color histograms, and pairwise classification systems, achieving disease classification through the extraction of color features from disease spots. Vaishnnave et al (Vaishnnave et al., 2019) employed the K-Nearest Neighbors (KNN) algorithm for crop leaf disease classification and identification, validated the feasibility of traditional machine learning methods in disease detection tasks. Nevertheless, these methods are confined to shallow feature extraction and exhibit poor adaptability to complex backgrounds and illumination variations, failing to meet the detection requirements in practical production environments.

In recent years, the rapid advancement of deep learning technology has provided novel solutions for tea disease detection (Yan et al., 2024). Convolutional Neural Networks (CNNs) have demonstrated remarkable advantages in crop disease recognition owing to their powerful automatic feature extraction capabilities, enabling the automatic learning of multi-level and high-order features from disease images, thereby overcoming the limitations of traditional handcrafted feature design (Soeb et al., 2023; Ahmad et al., 2023). Various deep learning models, including AlexNet, VGG, ResNet (He et al., 2016) and DenseNet, have been extensively applied to tea disease recognition tasks (Islam et al., 2022). For instance, Chen et al. (2019) proposed a CNN model named LeafNet, capable of automatically extracting deep feature information of tea leaf diseases from images, achieving end-to-end disease recognition. Li et al. (2022) integrated the SENet attention module into the DenseNet framework and employed transfer learning techniques for tea disease recognition, effectively improving the recognition accuracy under small sample conditions. These studies have demonstrated the effectiveness of deep learning in tea disease detection, establishing a foundation for intelligent tea plantation management.

With the continuous progress of object detection technology, from the You Only Look Once (YOLO) series (Redmon and Farhadi, 2018; Wang et al., 2024a; Redmon et al., 2016; Amir et al., 2025) to Real-Time Detection Transformer (RT-DETR) (Zhao et al., 2024b), numerous researchers have proposed improved schemes for crop disease detection tasks. Xue et al. (2023) proposed the YOLO-Tea model, enhancing the detection accuracy for tea diseases by improving the feature extraction network and loss function of YOLOv5. Lin et al. (2023) designed the TSBA-YOLO algorithm, introducing attention mechanisms and feature fusion strategies to strengthen the model’s recognition capability for tea diseases in complex backgrounds. Xia et al. (2024) proposed an improved lightweight YOLOv7 model based on MobileNeXt, enhancing the model’s ability to capture key details in disease images through a bi-level routing attention mechanism. Regarding RT-DETR improvements, Chen et al. (2024) proposed the RT-DETR-Tea model, optimized for multi-species tea bud detection tasks in unstructured environments, achieving an average precision of 96.3% in complex picking scenarios. Zhang et al. (2025a) proposed the SCORE-DETR network, improving the detection performance for small and occluded targets through a frequency aggregation network and Haar wavelet fusion module, achieving 54.0% AP on the citrus detection dataset. Kong et al. (2025) designed the Nextv2-DETR lightweight detection model, employing the ConvNeXtv2 backbone network and BiFormer attention mechanism, achieving lightweight deployment while maintaining high accuracy with only 16.9MB parameters. Wang et al. (2024b) proposed the FCHF-DETR model based on improved RT-DETR, optimizing the feature fusion structure for tomato leaf disease detection tasks and achieving a favorable balance between detection accuracy and speed. Zhang et al. (2025c) proposed the WMC-RTDETR model, achieving 97.7% mAP_50_ on tea disease and pest detection tasks while reducing parameters by 35.48% through the introduction of wavelet transform convolution and multi-scale multi-head self-attention mechanisms.

Although the aforementioned deep learning and object detection-based methods have achieved significant progress in tea disease detection tasks, they still face numerous challenges in practical complex agricultural environments. Firstly, tea diseases exhibit multi-scale and multi-morphological characteristics, with substantial variations in disease spot sizes, ranging from tiny early-stage lesions to large-scale latestage infections, making it difficult for traditional convolutional operations with fixed receptive fields to effectively capture disease features at different scales. Secondly, tea leaf images in natural environments present complex backgrounds with issues such as illumination variations, leaf occlusion, and similar texture interference, imposing higher requirements on the model’s global context modeling capability. Thirdly, although the Transformer architecture can achieve global context modeling, its O(n^2^) computational complexity results in excessive computational overhead, making it challenging to meet real-time detection requirements. Fourthly, the primary methods used for upsampling are bilinear interpolation and transposed convolutions; however, both methods lose features, which results in a lack of accurate localization of small lesions. There exists a gap between the lightweight design of the detection model and the high accuracy of the detection process, so developing an efficient-to-compute detection model that has both high detection accuracy and low model complexity continues to be a significant problem requiring resolution as soon as possible.

To overcome the previously mentioned challenges, this study employs a novel method for developing an efficient tea disease detection algorithm, referred to as DSA-DET, based on improvements of RT-DETR-R18 model architecture. The main contributions are as follows:

- Construction of a Tea Disease Detection Dataset: This research involved creating an AGTea database consisting of 5,972 images split into five separate disease categories: Blister Blight; Brown Blight; Gray Blight; Red Rust; and Healthy. The data was collected from a number of different angles and light conditions which were reviewed and annotated by experts thus addressing the shortfall of adequate samples in complex tea disease detection scenarios.
- Design of Dynamic Attention Spatial Pyramid Modeling Network (DASPMNet): A novel backbone network integrating dynamic attention mechanisms with spatial pyramid modeling is proposed. Through the Adaptive Feature Enhancement Block (AFEB), efficient extraction of multi-scale features is achieved, enhancing the representational capability for irregular disease spot morphology and complex texture features.
- Proposal of Hierarchical Spatial-Aware Transformer Encoder (HSATE): An efficient encoder module combining polarized linear attention with parallel spatial enhancement networks is designed, reducing computational complexity from O(n^2^) to O(n). Through the Multi-scale Adaptive Enhancement Module (MAEM), the discriminative capability for disease features at different scales is strengthened while ensuring global dependency modeling.
- Construction of Efficient Spatial-Channel Upsampling (ESCU): A lightweight upsampling module integrating depthwise separable convolution, channel shuffle, and spatial shift strategies is proposed, effectively addressing the information loss problem in traditional upsampling methods, improving feature reconstruction quality and disease boundary localization accuracy.

## 2 Materials and methods

### 2.1 Dataset

**Figure 1:**
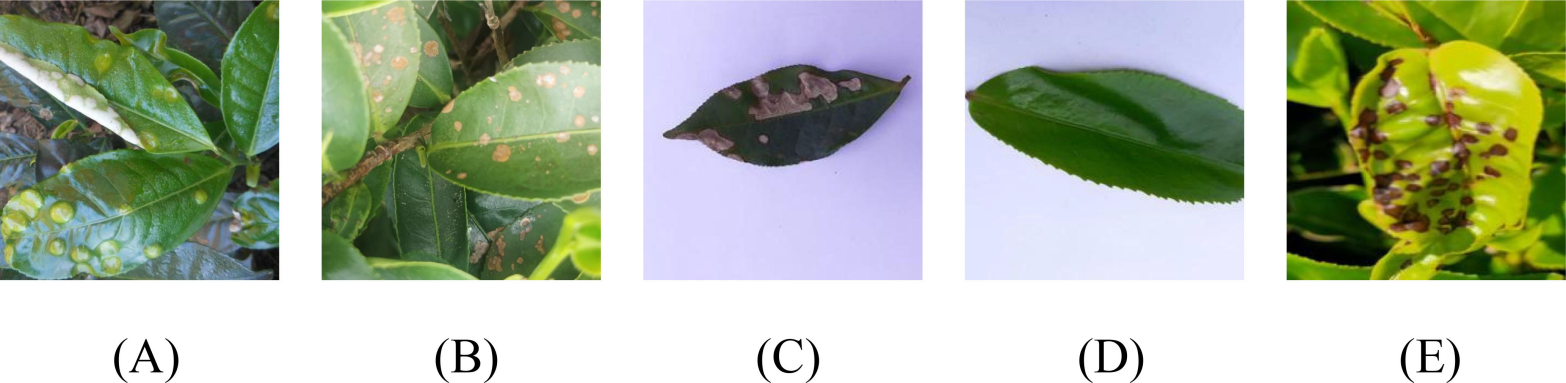
Examples of tea leaf disease images from the constructed AGTea dataset. **(A)** is Blister Blight, **(B)** is Brown Blight, **(C)** is Gray Blight, **(D)** is Healthy, **(E)** is Red Rust.

**Figure 2:**
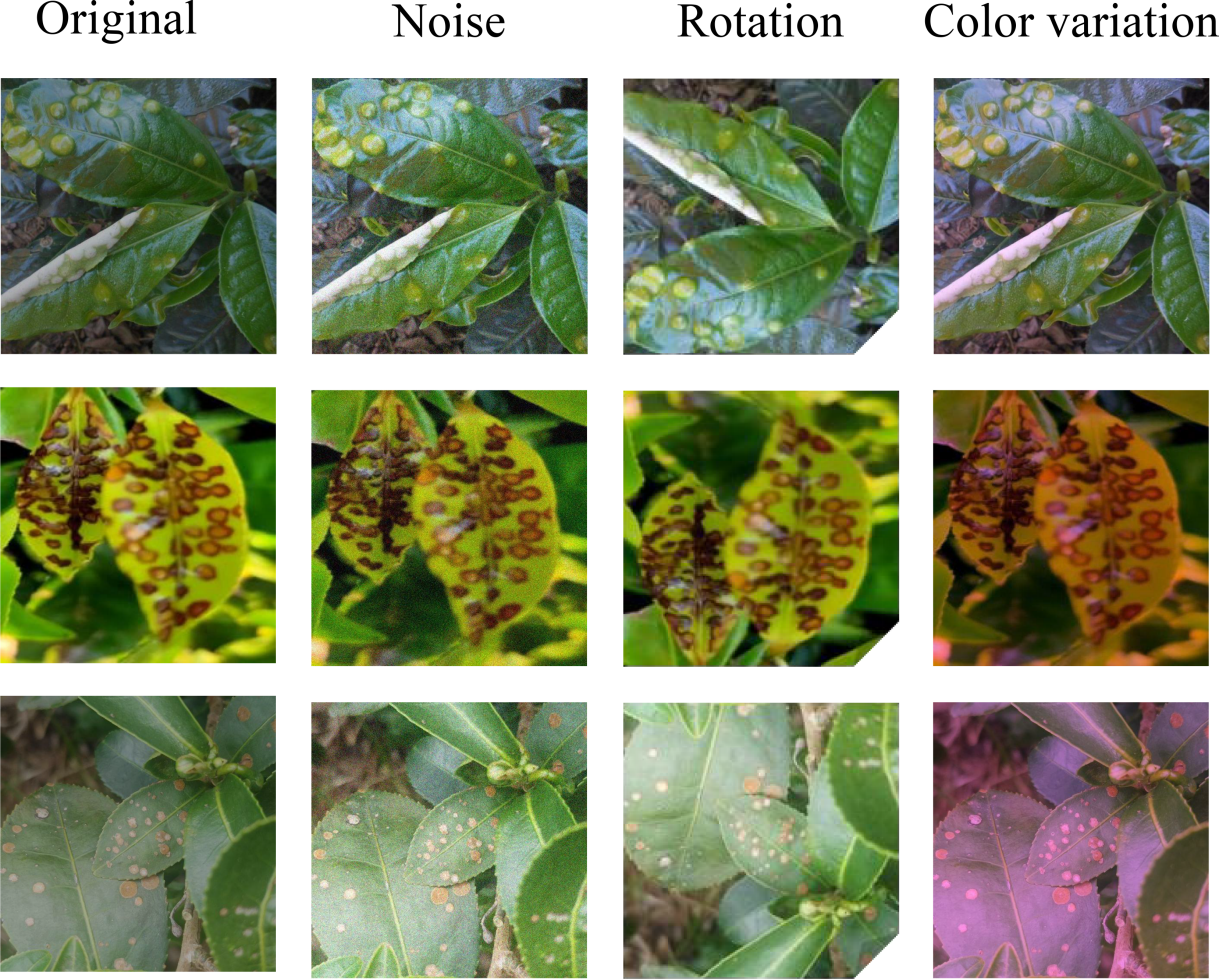
Examples of data augmentation techniques.

To address the limitations of existing tea disease datasets in evaluating complex scenarios, we constructed the AGTea dataset. Original images were collected from a Longjing tea plantation in Hangzhou, Zhejiang Province, between March 2023 and November 2024. Using an OPPO Find X6 Pro smartphone, we captured images in both natural field settings and standardized laboratory environments. The dataset comprises 4,778 original images spanning five conditions: Blister Blight (909 images), Brown Blight (1,029 images), Gray Blight (899 images), Red Rust (928 images), and Healthy leaves (1,013 images); typical samples are shown in **Figure 1**. Three volunteers independently annotated all images; these annotations were subsequently validated by industry experts and uniformly preprocessed to a resolution of 640 × 640 pixels. The original dataset was partitioned into training (2,986 images), validation (597 images), and test (1,195 images) sets at a 5:1:2 ratio. To simulate the variable shooting angles and environmental lighting encountered in practical applications, and to enhance model robustness and generalization, we applied data augmentation strategies—such as random rotation, hue transformation, and random noise addition—exclusively to the training set. This expanded the training set to 4,180 images; augmentation examples are shown in **Figure 2**. Consequently, the fully augmented AGTea dataset totals 5,972 images, shifting the final ratio of training, validation, and test sets to approximately 7:1:2.

**Figure 3:**
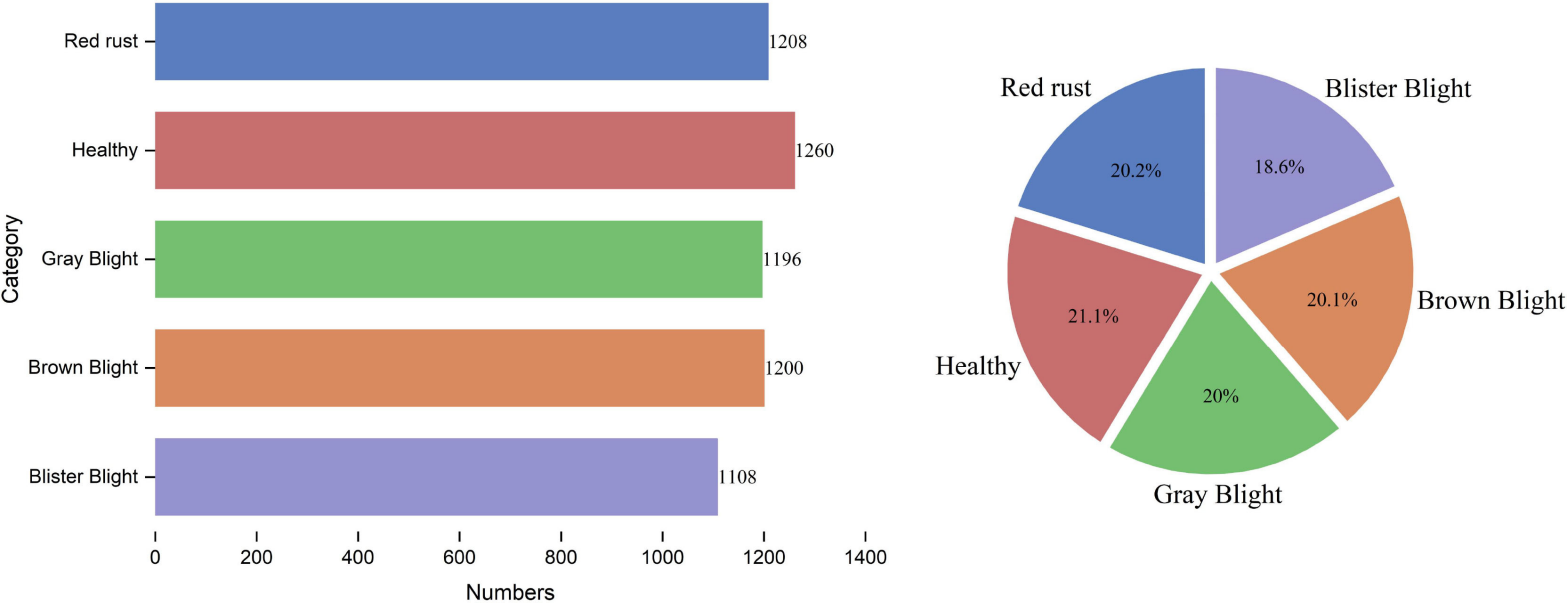
Distribution of tea leaf disease categories in the AGTea dataset.

**Figure 4:**
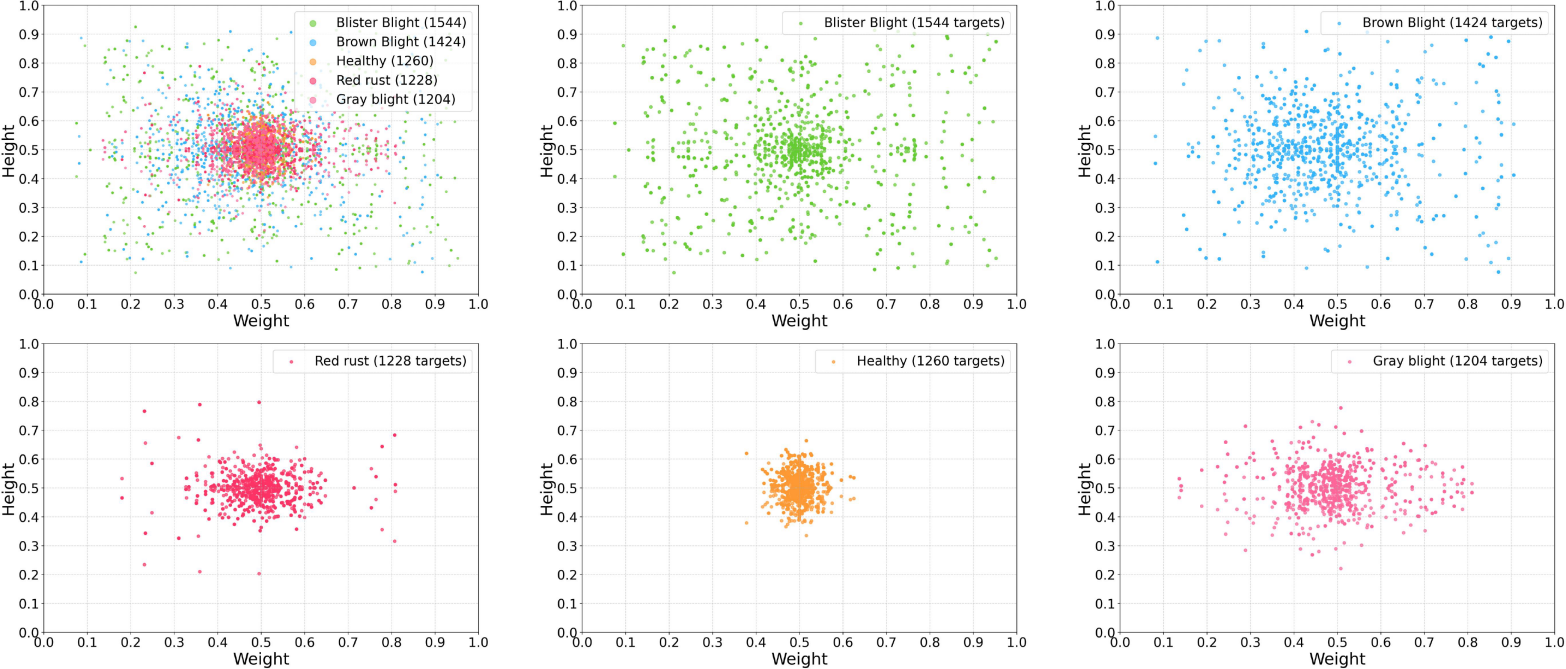
Feature distribution visualization of different tea leaf disease categories.

**Figure 5:**
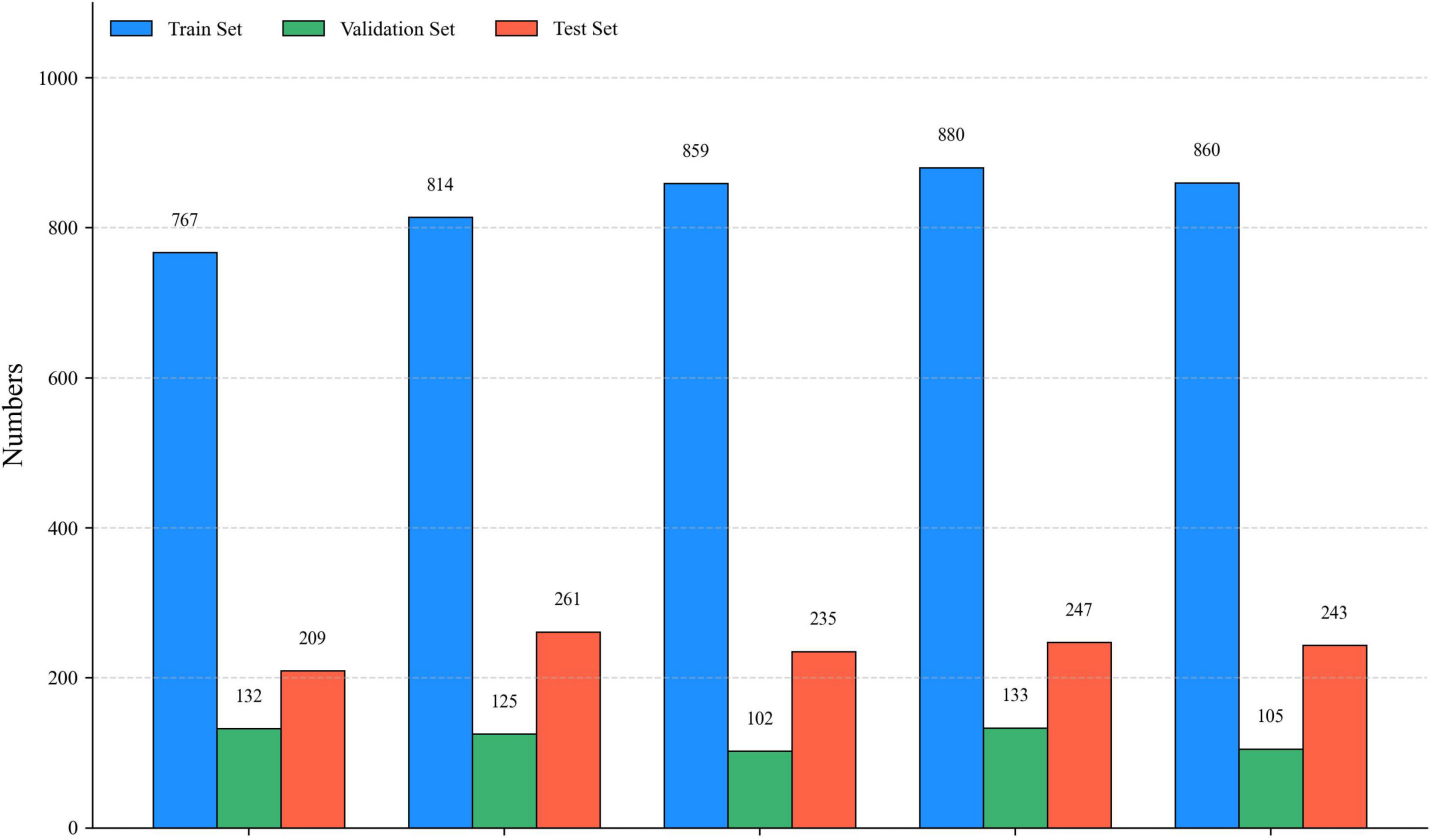
Visualization of dataset split across different tea leaf disease categories.

To comprehensively characterize the augmented dataset, we analyzed the sample distribution across the five target categories. As illustrated in **Figure 3**, this distribution is globally balanced, which facilitates the model’s robust extraction of disease-specific features. Furthermore, to evaluate the dimensional characteristics of the targets, we quantified the width and height of all bounding boxes. The resulting scatter plot, as shown in **Figure 4**, visually demonstrates the central tendency and dispersion of target sizes for each category. Finally, **Table 1** and **Figure 5** detail the exact sample allocation per category across the partitioned sets.

**Table 1:** Dataset split for each tea leaf disease category.

Target	Train set	Val set	Test set
Blister Blight	767	132	209
Brown Blight	814	125	261
Gray Blight	859	102	235
Healthy	880	133	247
Red Rust	860	105	243
Total	4180	597	1195

### 2.2 Overview of RT-DETR

**Figure 6:**
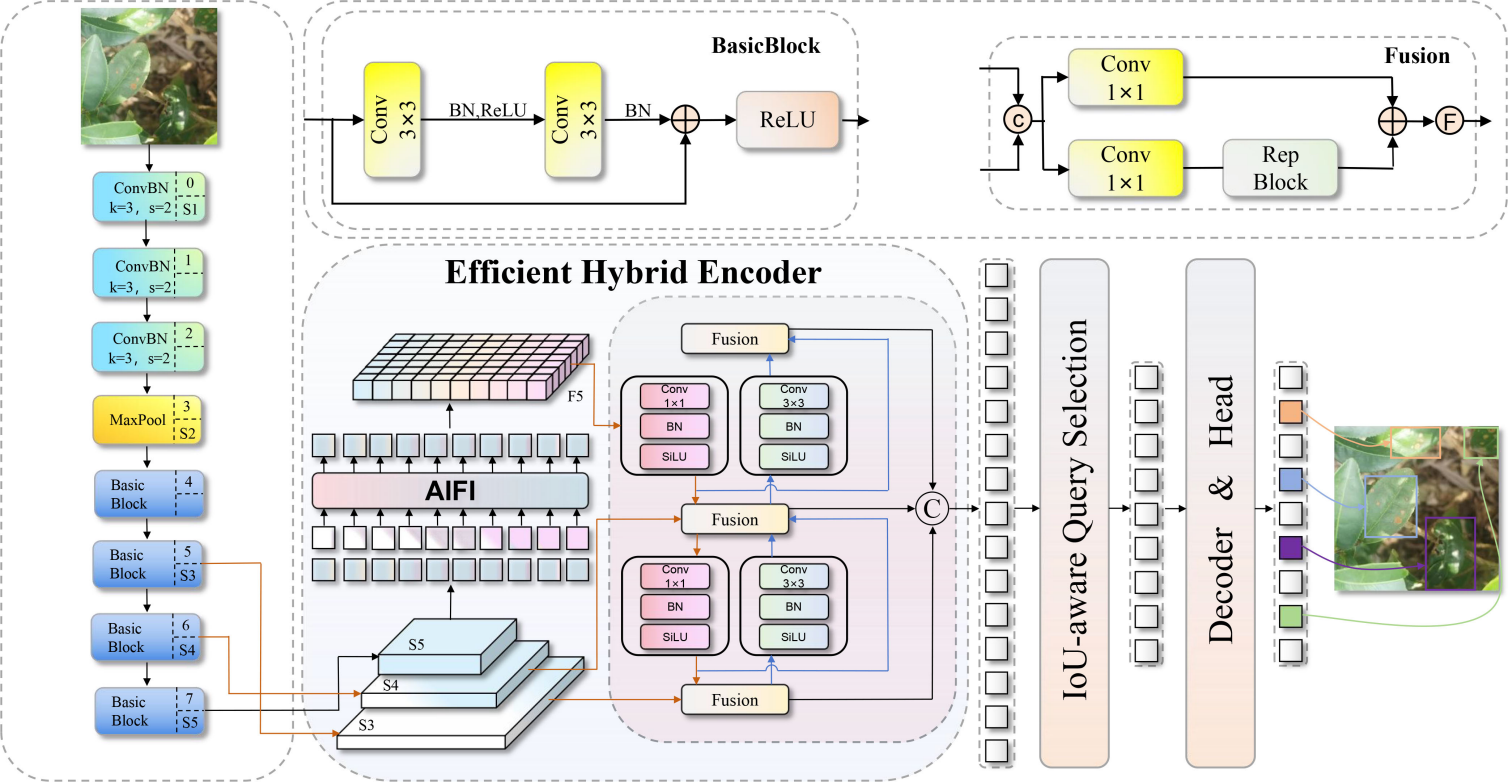
RT-DETR network structure diagram.

RT-DETR is the first Transformer-based architecture to achieve real-time, end-to-end object detection (Zhang et al., 2025b). By integrating an innovative hybrid encoder and an uncertainty-minimizing query selection strategy, it overcomes the excessive computational overhead and latency inherent in traditional DETR models. Unlike traditional YOLO-series detectors, RT-DETR simplifies the detection pipeline by eliminating the need for post-processing operations such as non-maximum suppression (NMS). Furthermore, its efficient encoder design substantially reduces computational complexity compared to the original DETR, conferring real-time detection capabilities to the Transformer architecture. The RT-DETR architecture comprises three primary components: a backbone network, an efficient hybrid encoder, and a Transformer decoder (Zhao et al., 2024a). The backbone extracts multi-scale feature maps from input images, typically employing HGNetV2 or ResNet-series architectures. Specifically, RT-DETR-R18 utilizes a lightweight ResNet18 backbone to minimize computational costs, with its structure shown in **Figure 6**. The hybrid encoder consists of two core modules: the Attention-based Intra-scale Feature Interaction (AIFI) and the CNN-based Cross-scale Feature-fusion Module (CCFM) (Chen et al., 2024). AIFI restricts Transformer self-attention operations to the highest-level semantic features to efficiently model global contextual dependencies. Concurrently, CCFM fuses multi-scale features via top-down and bottom-up bidirectional pathways, comprehensively integrating high-level semantic information with low-level spatial details. The decoder adopts a standard Transformer configuration equipped with an uncertainty-minimizing query selection strategy. It selects a fixed number of high-quality features from the encoder output as initial object queries (Li and Wang, 2025), which are then iteratively optimized using auxiliary prediction heads to yield precise detection results.

Despite this robust foundational framework, the standard RT-DETR-R18 exhibits inherent limitations when applied to tea disease detection. Primarily, the fixed convolutional kernels within its backbone struggle to adapt to the highly variable morphological characteristics of tea diseases, including diverse lesion sizes, irregular boundaries, and complex textural shifts. Furthermore, the relative shallowness of the RT-DETR-R18 network impedes the extraction of high-level semantic features, restricting its ability to distinguish subtle differences between diseased and healthy leaves. Finally, constrained local receptive fields limit the model’s capacity to identify disease patterns characterized by complex spatial relationships.

### 2.3 Establishment of the DSA-DET model

To address the aforementioned limitations of the base RT-DETR-R18 model, we propose the DSA-DET model. DSA-DET introduces three core modules specifically designed to overcome the base model’s constraints in agricultural applications: (1) DASPMNet: improved backbone network to enhance global context modeling capability; (2) HSATE: replaces the AIFI module in the encoder to achieve collaborative representation of global and local features; (3) ESCU: improves feature reconstruction and fusion capability in the upsampling stage.

**Figure 7:**
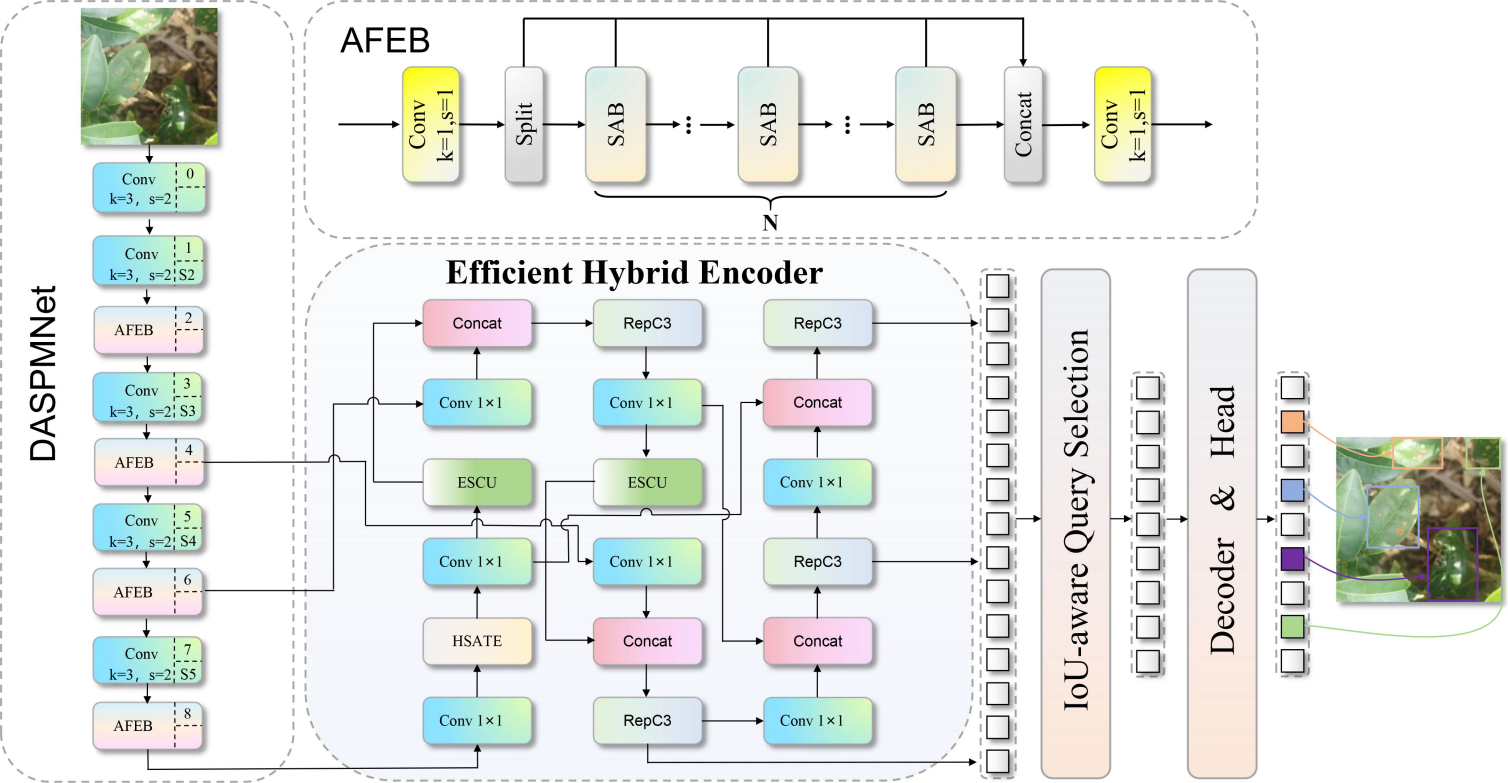
Overall architecture of the DSA-DET model.

The integration of these three modules follows a hierarchical feature processing paradigm: DASPMNet strengthens local feature representation at early stages with high spatial resolution, HSATE further captures long-range contextual dependencies at deep stages, and ESCU reconstructs spatial details during upsampling. This “local-to-global-to-detail” pipeline synergistically addresses multi-scale lesion detection and precise boundary localization. The overall structure of the improved network is shown in **Figure 7**.

#### 2.3.1 DASPMNet

**Figure 8:**
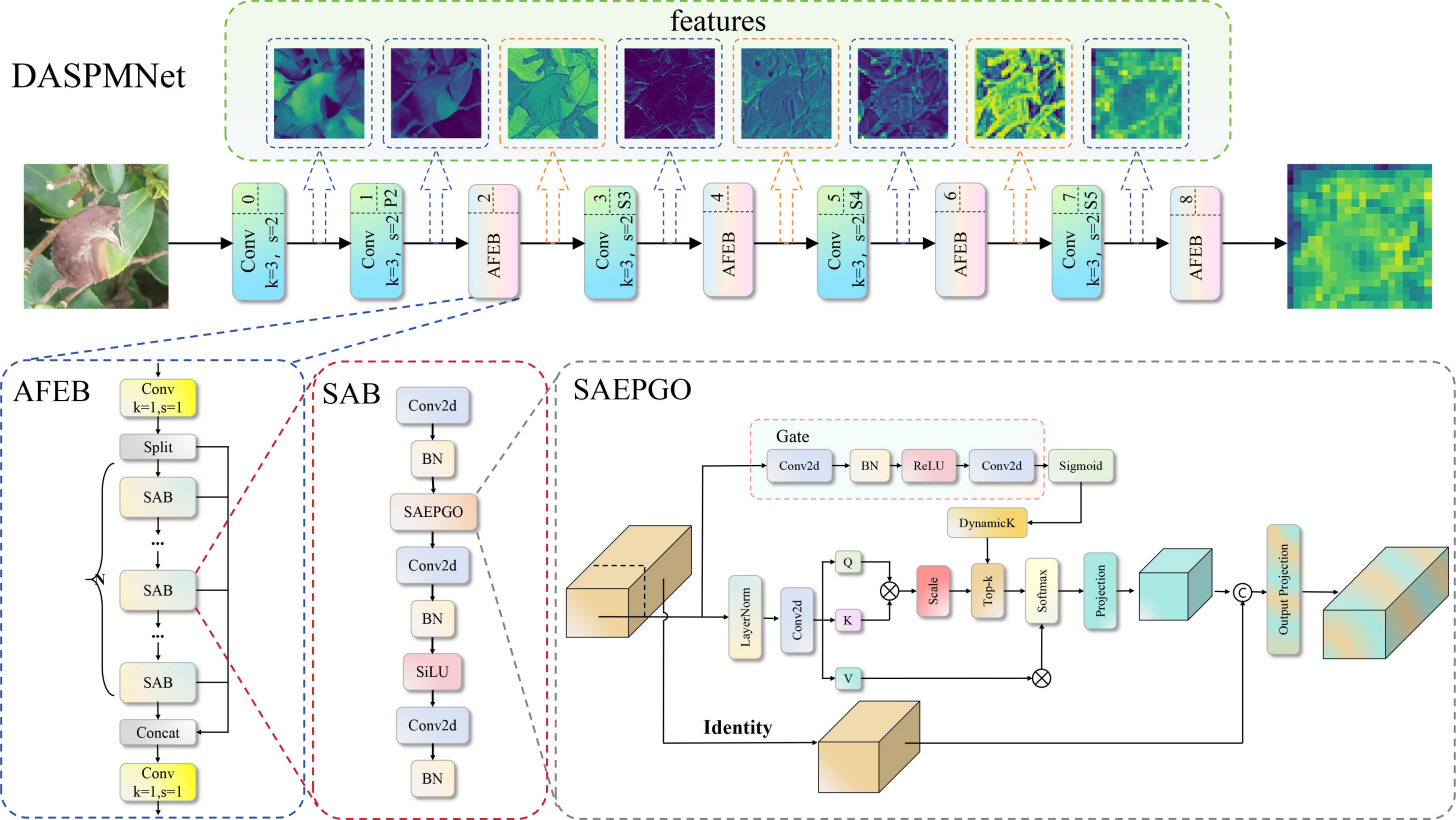
Structure of DASPMNet.

While ResNet-18 offers a lightweight architectural baseline for RT-DETR, it exhibits intrinsic representational deficiencies when deployed in the intricate landscape of tea disease detection. The morphology of tea diseases is characterized by substantial scale heterogeneity—ranging from minute, incipient lesions to extensive, late-stage infections—and high inter-class textural similarity, such as the subtle discrepancies between Blister Blight and Gray Blight. The static receptive fields inherent in ResNet-18’s convolutional kernels are fundamentally ill-equipped to accommodate these multi-scale spatial dynamics. Furthermore, the network’s constrained depth impedes the extraction of high-frequency textural fidelity, precipitating feature ambiguity amidst complex agricultural backgrounds. To surmount these impediments, we architected DASPMNet. By synthesizing dynamic attention mechanisms with spatial pyramid modeling, this architecture establishes a novel feature extraction paradigm capable of adaptively modulating receptive fields and constructing pixel-level long-range dependencies, thereby bolstering the model’s robustness against irregular lesion morphologies and intricate textural variations. The architecture of DASPMNet is illustrated in **Figure 8**.

The AFEB is designed as the core component of DASPMNet to replace conventional convolutional blocks. Its architecture draws upon the gradient flow optimization mechanism of the Cross Stage Partial (CSP) (Wang et al., 2020) topology and incorporates dynamic self-attention capabilities. To ensure a balanced distribution of computational efficiency and feature diversity during the initial stage of feature propagation, the input tensor is first divided into two orthogonal feature subspaces via 1 × 1 convolution, which reduces channel redundancy while enhancing feature diversity. mathematically expressed as Equation 1:

(1) $$\left\{F_{1},F_{2}\}=Split(Conv_{1\times 1}(X_{in}),\text{dim}=1\right)$$

where $X_{in}\in {\mathbb{R}}^{B\times C\times H\times W}$ denotes the input feature map, and $F_{1},F_{2}\in {\mathbb{R}}^{B\times (C/2)\times H\times W}$ represent the disentangled feature branches. Subsequently, cascaded Self-Attention Blocks (SAB) perform recursive feature aggregation to capture spatially dependent patterns of disease-related traits across different developmental stages. Each SAB encapsulates a Self-Attention Efficient Prompt Guide Operator (SAEPGO), which is conceptually derived from EPGO (Zou et al., 2025) and generates attention maps through dynamically created prompts, thereby effectively suppressing the interference of ambient noise on disease semantic saliency modeling. This recursive, deep feature interaction mechanism is expressed as Equation 2:

(2) $$F_{i}^{\left(l+1\right)}=SAB_{l}(F_{i}^{\left(l\right)}),\;l=1,2,\ldots ,n$$

In this iterative process, the output of a antecedent SAB serves as the prior for the subsequent unit, engendering a progressive flow of feature enhancement. To maximally preserve multi-level semantic hierarchies and restitute spatial granularity, AFEB employs a dense feature aggregation strategy at its terminus. By concatenating the primordial branches with the attentional features from all intermediate strata along the channel dimension, followed by cross-channel recombination via a terminal convolution layer, the model achieves an organic unification of local textural details and global semantic context as shown in Equation 3:

(3) $$Y_{final}=Conv_{1\times 1}(Concat([F_{1}^{1},F_{2}^{1},F_{1}^{2},F_{2}^{2},\ldots ,F_{2}^{\left(n\right)}]))$$

This architectural design not only effectively alleviates the vanishing gradient problem in deep networks but, through the adaptive allocation of dynamic weights, empowers DASPMNet to precisely delineate the complex boundaries and textural patterns of tea diseases, furnishing the detection head with high-fidelity feature maps rich in discriminative information.

#### 2.3.2 HSATE

**Figure 9:**
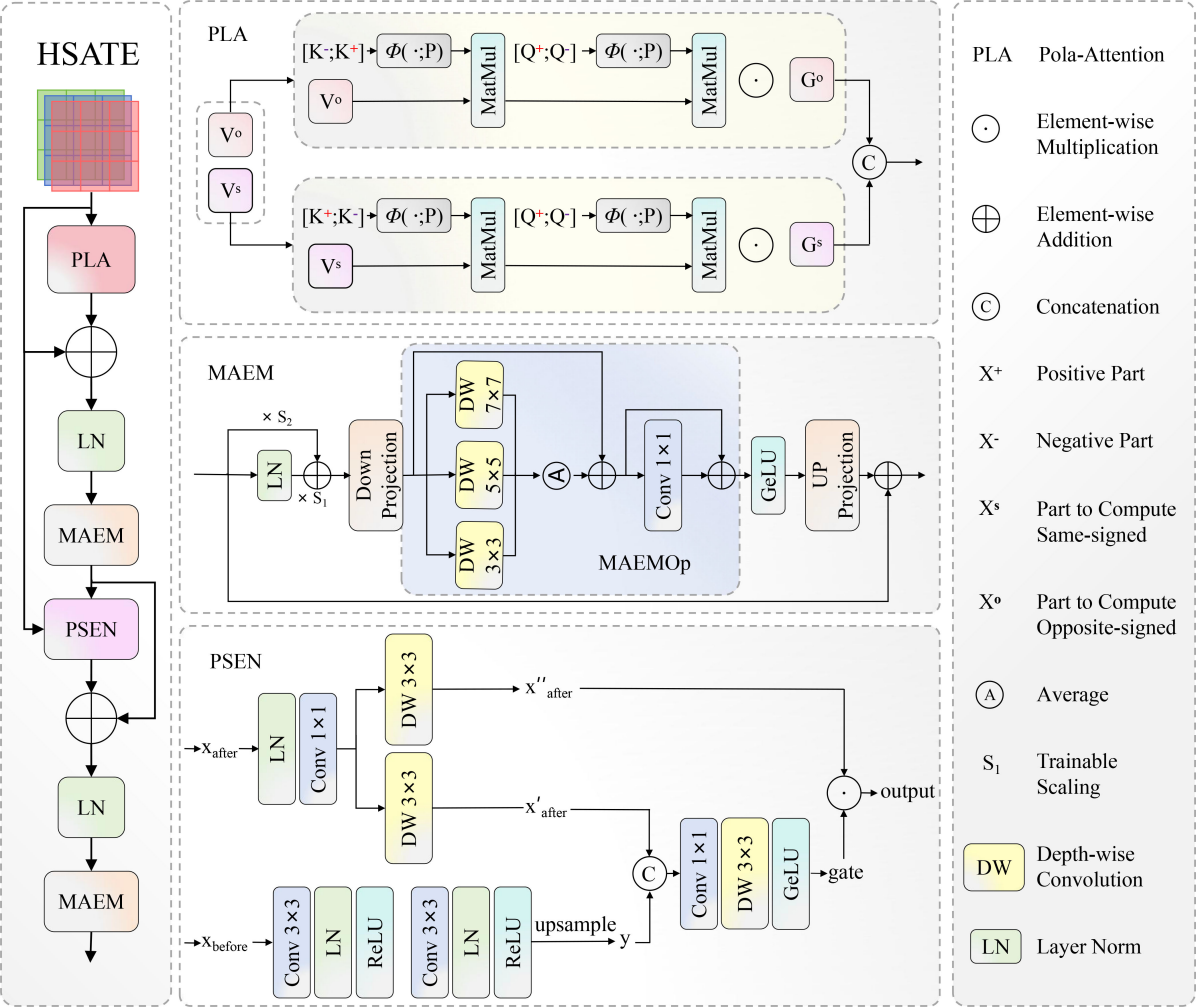
Structure of HSATE.

While the AIFI module in conventional RT-DETR introduces a global receptive field via the Transformer architecture, its core multi-head self-attention mechanism is constrained by a quadratic computational complexity of *O*(*n*^2^), imposing a severe computational bottleneck when processing high-resolution imagery of tea diseases. To preserve the pixel-level fidelity requisite for distinguishing incipient, minute lesions (such as early-stage Red Rust spots), high image resolution is indispensable; however, this directly precipitates prohibitive GPU memory consumption and latency in standard architectures. Furthermore, the canonical Feedforward Network (FFN) within AIFI tends to dissipate spatial context during feature transformation, rendering the model inept at modeling the spatial dependency of disease distribution across the leaf surface. Addressing these impediments, we constructed HSATE. As shown in **Figure 9**, aiming to achieve global modeling of high-resolution features at linear computational cost, while simultaneously compensating for the inherent deficiencies of standard Transformers in capturing fine-grained pathological features through multi-scale spatial enhancement mechanisms.

The design philosophy of HSATE adheres to a hierarchical flow of “linear global modeling—multiscale calibration—spatial detail reconstruction.” Initially, to circumvent the computational barrier without compromising long-range dependencies, we incorporate polarized linear attention (Meng et al., 2025) (PLA). The standard self-attention mechanism computes according to Equation 4:

(4) $$Attention(Q,K,V)=softmax(\frac{QK^{T}}{\sqrt{d_{k}}})V$$

where $Q,K,V\in {\mathbb{R}}^{N}\times d$ denote query, key, and value projections with *N* representing the sequence length, and *d_k_* denotes the dimension of the key vectors. This incurs *O*(*N*^2^) computational complexity due to the *QK^T^* matrix multiplication. In contrast, PLA reformulates the attention computation by leveraging the kermel function technique with sign-splitting strategy, transforming the quadratic complexity to linear. Specifically, PLA decomposes the original attention into positive and negative polarities as defined in Equation 5:

(5) $$PLA(Q,K,V)={\left[\phi {\left(Q^{+}\right)}^{T}\cdot (\frac{1}{N}\sum_{i=1}^{N}\phi {\left(K_{i}^{+}\right)}^{T}V_{i})\right]}_{+}+{\left[\phi {\left(Q^{-}\right)}^{T}\cdot (\frac{1}{N}\sum_{i=1}^{N}\phi {\left(K_{i}^{-}\right)}^{T}V_{i})\right]}_{-}$$

where $\phi (\cdot )=\text{ReLU}{\left(\cdot \right)}^{\alpha }$ is the kernel function with learnable power parameter *α*. $Q^{+}/Q^{-}$ and $K^{+}/K^{-}$ represent the positive/negative polarity components, respectively. By furst camputing the pooled keyvalue representation $\frac{1}{N}K^{T}V\in {\mathbb{R}}^{d\times d}withO(N)$ complexity, and then performing $Q\cdot (K^{T}V)$ with O(N) complexity the overall computational complexity is reduced to O(N). Subsequently, the feature stream undergoes deep interaction within our designed MAEM and parallel spatial enhancement network (PSEN). MAEM is responsible for immediate multi-scale adaptive calibration following attention, while PSEN supersedes the traditional FFN to restitute compressed spatial details. The forward propagation of the entire encoder is formalized as an alternating enhancement mathematical paradigm as shown in Equations 6 and 7:

(6) $$Z_{attn}=MAEM_{1}(LN(X+Dropout(PLA(X))))$$

(7) $$Z_{out}=MAEM_{2}(LN(Z_{attn}+Dropout(PSEN(Z_{attn},X))))$$

where $X\in {\mathbb{R}}^{B\times HW\times C}$ represents the input feature tokens, and *LN*(·) signifies Layer Normalization. *PLA* refers to the Polarized Linear Attention mechanism, while *PSEN* represents the Parallel Spatial Enhancement Network. *MAEM*_1_ and *MAEM*_2_ denote the Multi-scale Adaptive Enhancement Modules, which are strategically deployed to enforce scale-invariant feature refinement before and after the spatial mixing stage.

To rectify the deficiency of spatial structure loss inherent in traditional feedforward networks during feature mapping, we designed PSEN. Adopting a dual-branch parallel topology, this module aims to achieve a complementary fusion of “local granularity” and “global spatial context.” Unlike the singular mapping of standard FFNs, PSEN initially expands dimensions via 1 × 1 convolution and subsequently bifurcates the feature stream. The spatial branch utilizes average pooling downsampling to extract abstract global context descriptors, which are processed through a convolutional sequence before resolution restoration via upsampling; conversely, the main branch preserves the original fine-grained features. The two are dynamically recombined via a gating mechanism during the fusion stage, enabling the network to implicitly learn the spatial distribution priors of diseases, effectively capturing pathological features with specific spatial dependencies, such as the propagation patterns of Blister Blight along leaf veins, as defined in Equations 8 and 9:

(8) $$F_{spatial}=Upsample(ConvSeq(AvgPool(X_{input},s=2)),s=2)$$

(9) $$F_{out}=Conv_{1\times 1}(GELU(DWConv(Fusion(Concat(F_{spatial},F_{1})))))⊙F_{2}$$

where *F_spatial_* represents the global context features from the spatial branch, *s* = 2 represents the sampling multiple, *F*_1_ and *F*_2_ are main branch feature components, *Fusion* represents feature fusion convolution, and ⊙ represents the gating mechanism.

Confronting the substantial scale variation of tea disease lesions—ranging from pin-point spots to extensive patches—single-scale convolutional kernels prove inadequate. Consequently, we designed MAEM. Embedded with the core component MAEMOp, this module employs a three-path parallel depthwise separable convolution array (kernel sizes of 3 × 3,5 × 5,7 × 7) to construct a multi-granular feature capture field. This structure not only adaptively aggregates feature responses across varying receptive fields but also introduces learnable scaling parameters *γ* and mixing parameters *γ_x_* to dynamically modulate the intensity of feature enhancement. Its core operation is defined as Equations 10 and 11:

(10) $$MAEMOp(X)=X+\frac{1}{3}\sum_{k\in \{3,5,7\}}DWConv_{k\times k}(X+Conv_{1\times 1}(X))$$

(11) $$MAEM(X)=X+Conv_{1\times 1}(Dropout(GELU(MAEMOp(Conv_{1\times 1}(X\cdot \gamma +LN(X)\cdot \gamma_{x})))))$$

where *γ* and *γ_x_* are learnable feature scaling and mixing parameters, and *MAEMOp* represents the multi-scale adaptive enhancement operation.

Through this design, MAEM dynamically adjusts sensitivity to diseases of different scales at various stages of feature encoding, ensuring that minute lesions are not overlooked while large-area pathological features remain cohesive. Through the organic synergy of the aforementioned modules, HSATE theoretically achieves a dual leap in computational efficiency and feature expressiveness, not only reducing GPU memory footprint but also enhancing the detection precision and localization accuracy of various tea diseases against complex field backgrounds.

#### 2.3.3 ESCU

**Figure 10:**
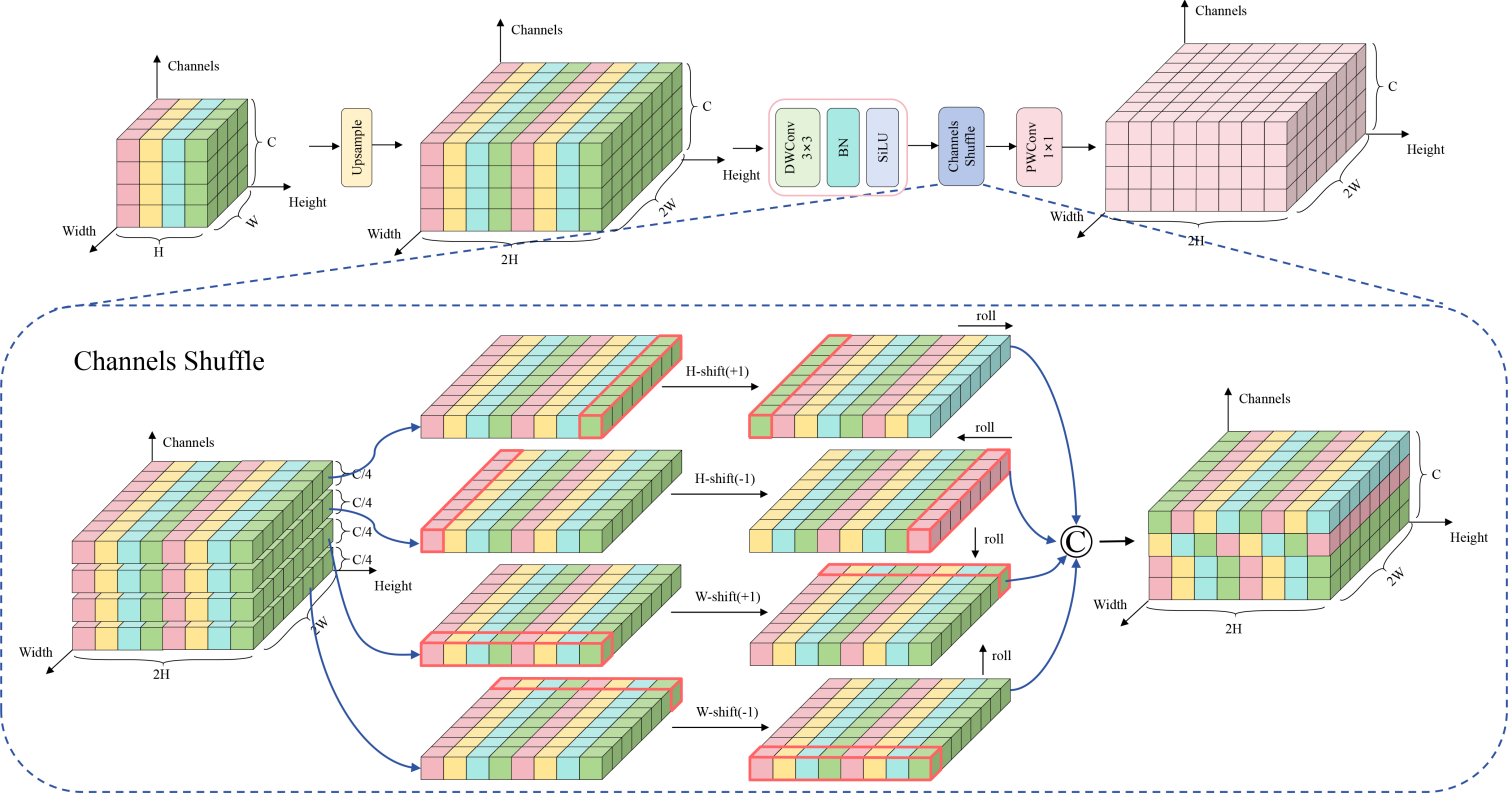
Structure of ESCU.

Traditional upsampling methods, such as bilinear interpolation and transposed convolution, exhibit significant limitations in object detection tasks. These methods frequently incur information loss and feature degradation during the rendering process, leading to the deterioration of spatial details—a particularly critical issue for tea disease detection tasks that demand precise localization of small disease spots. Furthermore, existing upsampling techniques lack mechanisms for effective cross-channel information interaction, thereby constraining the model’s representational capacity for complex disease features. To address these challenges, this paper proposes an efficient upsampling module named ESCU. As illustrated in **Figure 10**, ESCU adapts the ShiftChannelMix (Gao et al., 2025) and integrates it with depthwise separable convolutions and a tailored spatial shift mechanism. This synergistic combination substantially enhances feature representation while preserving spatial details.

ESCU follows multiple stage in processing the features. First, it upsamples the spatial resolution of the input feature map by 2x through nearest neighbor interpolation and then extracts features via depthwise separable convolution, which reduces the computation cost. The main novelty of the module lies in the adaptive channel shuffle and spatial shift strategy and the channel shuffle can be mathematically expressed as Equation 12:

(12) $$X_{shuffle}=R(T(R(X,(B,G,C/G,H,W),\;(0,2,1,3,4))),(B,C,H,W))$$

where $X\in {\mathbb{R}}^{B\times C\times H\times W}$ represents the input feature map, R denotes Reshape and T denotes Transpose, with *B*, *C*, *H*, *W* respectively representing batch size, number of channels, height, and width, and *G* being the number of groups. This operation promotes information exchange between different feature groups by reordering channel sequences. Subsequently, the spatial shift mixing mechanism divides channels into four subsets and applies shift operations in different spatial dimensions. The mathematical expression is shown in Equation 13:

(13) $$X_{shift}=Concat[Roll(X_{1},s,dim=2),Roll(X_{2},-s,dim=2),Roll(X_{3},s,dim=3),Roll(X_{4},-s,dim=3)]$$

where *X_i_* represents the *i*-th channel subset, *s* is the shift step size, and *Roll* (·*,s*,dim) represents the circular shift operation on the specified dimension. This design enables the model to implicitly learn local spatial context relationships, enhancing perception capability for disease boundaries while maintaining low computational overhead.

Through the above innovation of design, ESCU has obtained performance advantage in the task of detecting tea disease. By obtaining high-quality feature reconstruction via this module, the detection of disease targets is improved. The shuffling of channels promote cross-channel flow of information, enhances the discriminability of features, and the spatial shift strategy establishes local spatial dependencies thereby improving the model’s precise location capability for the boundary of the disease. And the synergistic effect of these two technical innovations enables ESCU to provide important technical support for the performance improvement of the RT-DETR model on tea disease detection tasks while guaranteeing real-time performance.

## 3 Experimental results and analysis

### 3.1 Model evaluation metrics

This paper employs precision(P), recall(R), F1 score, mAP_50_, mAP_50:95_, model parameters, giga floating point operations per second (GFLOPs) and frames per second (FPS) as assessment metrics. The following are their calculation formulas (Equations 14, 15):

(14) $$Precision=\frac{TP}{TP+FP}$$

(15) $$Recall=\frac{TP}{TP+FN}$$

(16) $$F1-score=\frac{2PR}{P+R}$$

(17) $$mAP=\frac{1}{n}\sum_{i=1}^{n}AP_{i}$$

where TP (true positive) denotes correctly predicted positive samples, correctly predicted negative samples by TN (true negative), mistakenly predicted positive samples by FP (false positive), and incorrectly predicted negative samples by FN (false negative). The F1 score serves as the harmonic mean of precision and recall, as shown in **Equation 16**. mAP_50_ represents the average precision at an IoU threshold of 0.5, calculated as the area under the precision-recall curve, as shown in **Equation 17**. mAP_50:95_ considers IoU values between 0.5 and 0.95. FPS denotes the quantity of frames the model can process per second; GFLOPs denote billion floating-point operations required for computation; model size and complexity are key reference metrics for lightweight models, which this paper expresses in megabyte units.

### 3.2 Experimental framework and model parameter configurations

**Figure 11:**
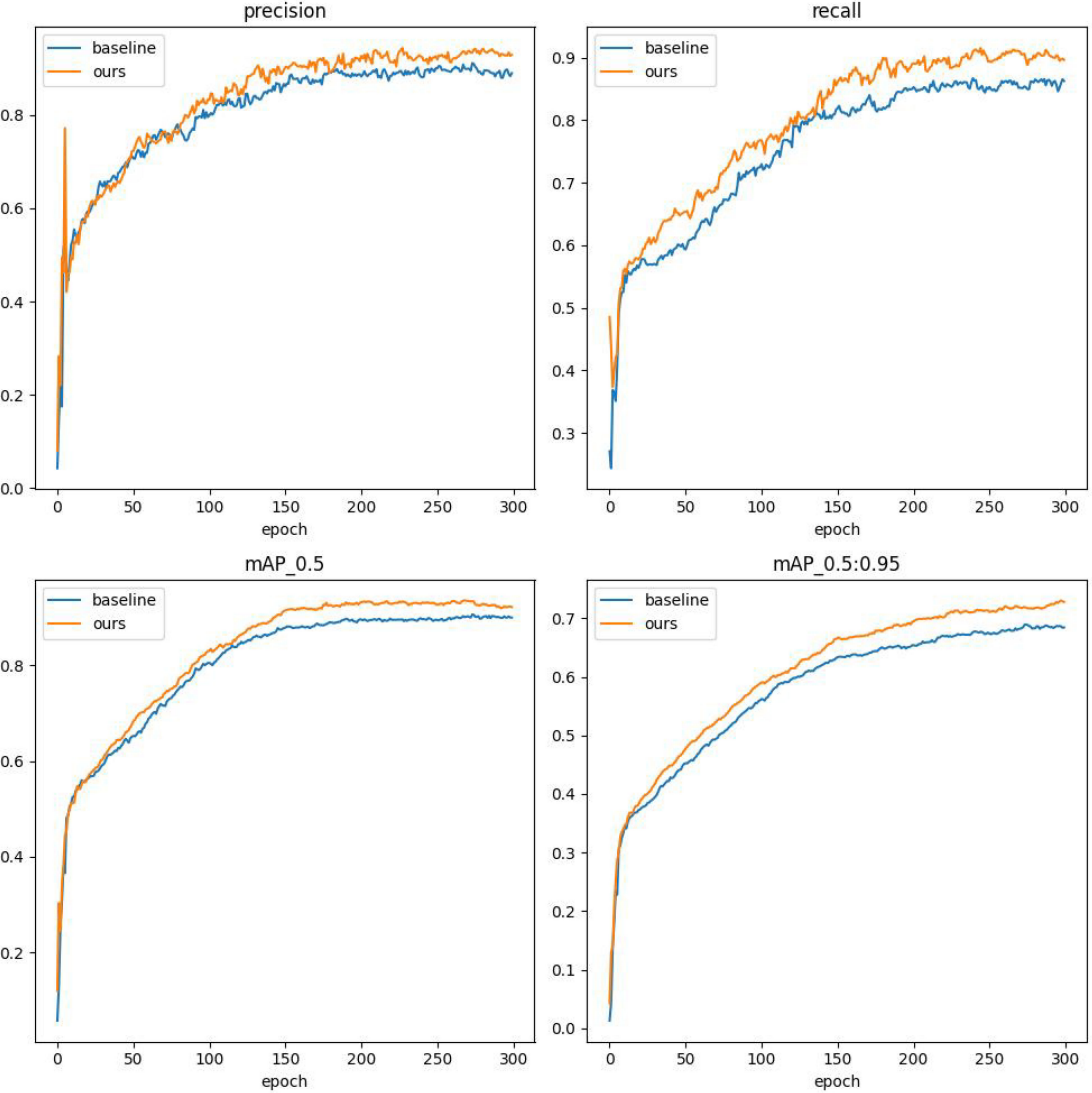
Performance comparison radar chart of comparative experiment results.

All experiments in this research utilize the PyTorch deep learning framework, with Python as the programming language. The primary configuration of the experimental computer is presented in **Table 2**. The main parameter configurations of the model are presented in **Table 3**. All experiments were conducted under identical configurations. The training curves of precision, recall, mAP50, and mAP50:95 for both the baseline and DSA-DET are illustrated in **Figure 11**.

**Table 2:** Experimental environment and configuration.

Experimental environment	Parameter/Version
Operating System	Ubuntu 22.04
GPU	NVIDIA RTX 3090
CPU	Intel Xeon Gold 6330 CPU (14 vCPUs, 2.00 GHz)
CUDA	11.8
PyTorch	2.4.1
Python	3.8.20

**Table 3:** Main parameters of experiment.

Train parameter	Value
Input size	640
Batch size	4
Maximum epochs	300
Momentum	0.9
Learning rate	0.0001
Weight decay	0.0001
Warm-up epochs	2000
Optimizer	AdamW

### 3.3 Comparison of results for different backbone networks

As the feature extraction foundation of object detection models, the backbone network architecture choice has a decisive impact on model performance. To comprehensively evaluate performance differences of different backbone networks, this research systematically compared ResNet18, ResNet34, ResNet50, ResNet101, and HGNetV2. Experiments conducted comprehensive analysis from two dimensions: model complexity and detection performance. Model complexity was measured by number of parameters (Parameters) and floating-point operations (FLOPs), while detection performance used mean average precision (mAP_50:95_) as the evaluation metric. **Table 4** presents detailed comparison results of each backbone network under the above metrics, providing quantitative basis for subsequent network selection.

**Table 4:** Analysis of the outcomes for various backbones.

Backbone	Parameters	GFLOPs	mAP_50:95_
HGNetV2	31.9M	103.5G	70.16
ResNet101	74.7M	247.1G	67.15
ResNet50	41.9M	129.6G	71.5
ResNet34	31.1M	88.8G	68.8
ResNet18(Baseline)	19.9M	57.0G	68.94

ResNet18 is the lightweight network in the ResNet family. Compared to other networks, its structure is more concise with lower complexity. From experimental results, we can see that ResNet18 demonstrates excellent cost-effectiveness advantages in tea disease detection tasks. Although its parameter count is only 19.9M and computational complexity is the lowest among all tested models at only 57.0 GFLOPs, its mAP_50:95_ reaches 68.94%, even slightly superior to ResNet34 with more parameters. This indicates that ResNet18’s network architecture is highly matched with the complexity of tea disease detection tasks. Compared to the best-performing ResNet50, ResNet18 shows only a 2.56% decrease in accuracy, but with a 52.5% reduction in parameters and 56% decrease in computation, improving the model’s lightweight degree while ensuring detection accuracy. This makes ResNet18 the optimal backbone network choice in this research, more suitable for implementation on resource-limited edge devices and real-time detection scenarios.

### 3.4 Ablation experiments

To evaluate the effectiveness of the three core innovative modules in the DSA-DET algorithm, we designed detailed ablation experiments. Using RT-DETR-R18 as the baseline model, we employed the controlled variable method to separately verify the independent contributions and combined effects of DASPMNet, HSATE, and ESCU. Experiments covered single modules, dual module combinations, and complete model performance comparisons, comprehensively evaluating each component’s impact on detection accuracy, model complexity, and inference efficiency. Experimental results are shown in **Table 5**.

**Table 5:** Results of the ablation experiments.

Methods	DASPMNet	HSATE	ESCU	P	R	mAP_50_	mAP_50:95_	GFLOPs	Parameters	FPS
Baseline				0.9117	0.8669	0.9066	0.6894	57G	19.9M	80.3
Improvement 1	✓			0.9283	0.8818	0.9212	0.7057	50G	13.6M	81.8
Improvement 2		✓		0.9267	0.8795	0.9186	0.7048	51.7G	21.6M	81
Improvement 3			✓	0.9187	0.8735	0.9126	0.6968	38G	20M	85.3
Improvement 4	✓	✓		0.9389	0.8892	0.9289	0.7174	50.8G	15.3M	82.4
Improvement 5	✓		✓	0.9305	0.8830	0.9227	0.7092	51.1G	13.7M	83.2
Improvement 6		✓	✓	0.9254	0.8771	0.9165	0.7048	38.8G	21.7M	88
Ours	✓	✓	✓	0.9473	0.8965	0.9368	0.7276	51.9G	15.4M	71.5

Ablation experimental results deeply validate the specific functional effects of each module. DASPMNet through its dynamic weight allocation and adaptive feature aggregation mechanisms, effectively demonstrates the capability to achieve model lightweighting while maintaining detection accuracy. When used alone, mAP_50_ improved by 1.46%. More importantly, parameters were compressed by 31.7%, fully validating this module’s superiority in redundant parameter elimination and efficient feature extraction. HSATE specifically designed for the multi-scale feature distribution characteristics of tea diseases, incorporating PLA to replace the standard AIFI module. Ablation experiments demonstrate that integrating HSATE alone reduces computational overhead from 57.0G to 51.7G GFLOPs, representing a 9.3% decrease, while enhancing small target detection capability in complex backgrounds. mAP_50_ improved to 91.86%, with particularly obvious improvement in the mAP_50:95_ metric, validating this module’s effectiveness in multi-scale feature fusion and fine-grained feature preservation. ESCU through shared convolution kernels and unified branch design, experimental results prove its outstanding performance in computational efficiency optimization. GFLOPs decreased by 33.3%, and inference speed improved to 85.3 FPS, demonstrating this module’s innovative value in reducing redundant computations and optimizing inference processes. This comprehensively validates the significant improvement effects of each module’s synergistic action in three dimensions: precision improvement, model compression, and efficiency optimization.

### 3.5 Comparative experiments

**Figure 12:**
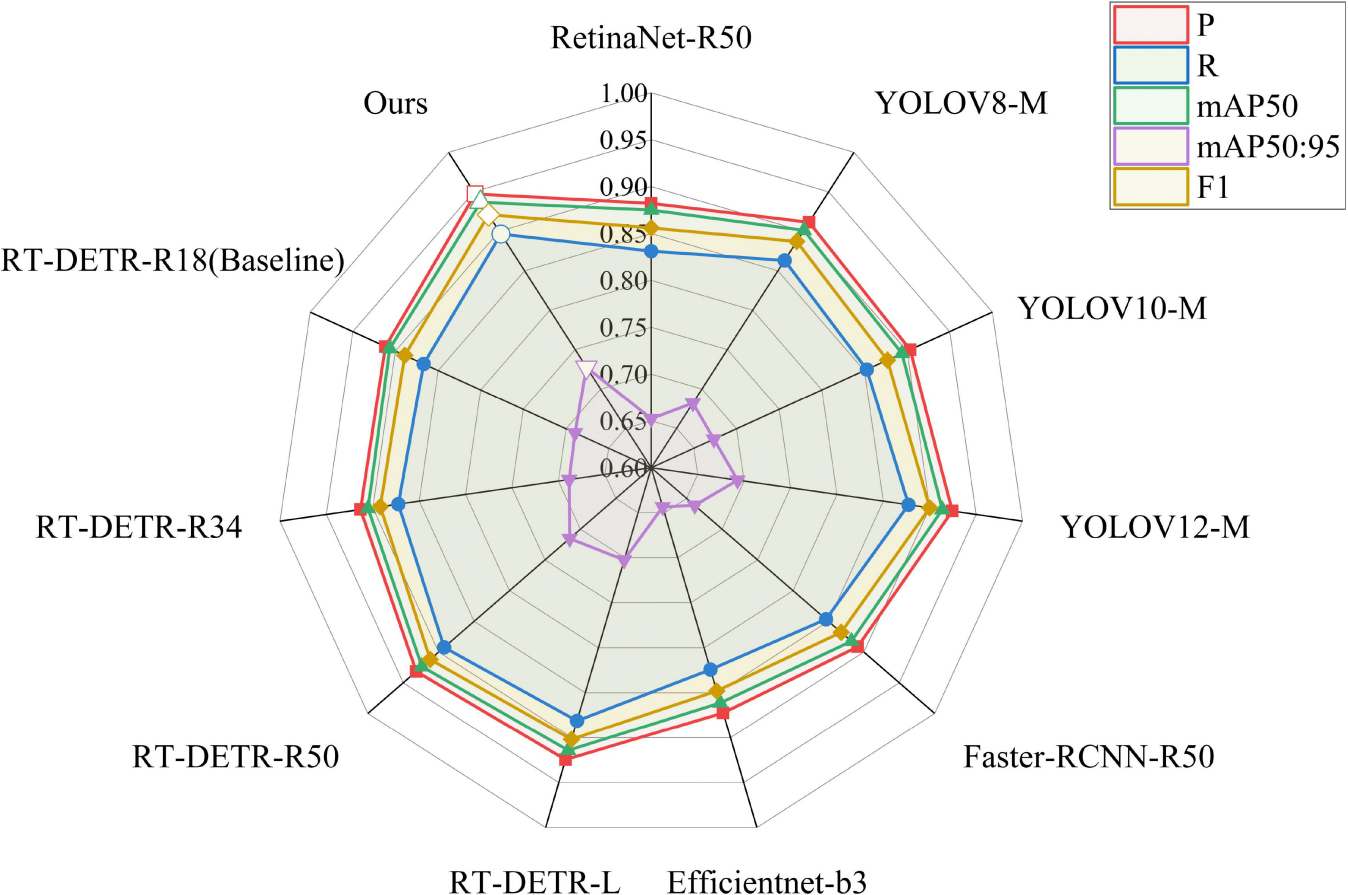
Comparison of evaluation metrics between the baseline and ours.

To validate the effectiveness of our designed DSA-DET, we performed comparative experiments with different SOTA models. Experimental results are presented in **Table 6**, and **Figure 12** compares the performance distribution of major detection models through radar chart format. For single-stage object detectors, we compared YOLO variants (YOLOv8/10/12-M) and RetinaNet (Lin et al., 2017). For two-stage object detectors, we compared Faster R-CNN (Ren et al., 2016) and Efficientnet (Tan and Le, 2019). For Transformer-based object detectors, we selected the RT-DETR series. Among them, R18/R34/R50 denote ResNet-18/34/50 backbone networks, -M indicates medium-scale versions, and b3 signifies EfficientNet’s compound scaling coefficient. All models were trained and tested on the same tea disease dataset to ensure fairness and comparability of experimental results.

**Table 6:** Results of multi-model comparison experiments.

Model	P	R	mAP_50_	mAP_50:95_	F1	GFLOPs	Parameters	FPS
*One-stage object detector*								
RetinaNet-R50	0.8821	0.8312	0.875	0.6523	0.856	210G	36.15M	56.6
YOLOV8-M	0.9114	0.8631	0.9011	0.6821	0.887	78.7G	25.84M	121.4
YOLOV10-M	0.9035	0.8527	0.894	0.6735	0.877	63.5G	16.46M	105.3
YOLOV12-M	0.9244	0.8772	0.9136	0.6931	0.900	67.2G	20.11M	106.2
*Two-stage object detector*								
Faster-RCNN-R50	0.8913	0.8467	0.8825	0.6613	0.868	208G	41.39M	51.3
Efficientnet-b3	0.8725	0.8243	0.8613	0.6435	0.848	62.5G	18.52M	59.7
*DETR-based object detector*								
RT-DETR-L	0.9246	0.8813	0.9142	0.7025	0.902	103.4G	31.9M	64.3
RT-DETR-R50	0.9316	0.8923	0.9237	0.7152	0.912	129.6G	41.9M	51.4
RT-DETR-R34	0.9133	0.8725	0.9051	0.6884	0.892	88.8G	31.1M	60.3
RT-DETR-R18 (Baseline)	0.9117	0.8669	0.9066	0.6894	0.889	57G	19.9M	80.3
Ours	0.9473	0.8965	0.9368	0.7276	0.921	51.9G	15.4M	71.5

Experimental results show that different types of object detection models exhibit significant differences in performance on tea disease detection tasks. Among single-stage detectors, YOLOv12-M performed relatively well with mAP_50_ of 91.36%, but there is still room for improvement. RetinaNet-R50, as a classic single-stage detector, showed relatively lower performance with mAP_50_ of 87.5%. Among two-stage detectors, Faster-RCNN-R50 achieved 88.25% mAP_50_ through its structure, but its 208G GFLOPs computation and 51.3 FPS speed limit practical applications. Among DETR-based detectors, RT-DETR-L achieved 91.42% mAP_50_, demonstrating the advantages of Transformer architecture, but with a relatively large parameter count of 31.9M.

In comparison, our proposed DSA-DET in tea disease detection tasks achieves excellent performance with precision of 94.73%, recall of 89.65%, and mAP_50_ of 93.68%, representing improvements of 3.56%, 2.96%, and 3.02% respectively over the baseline RT-DETR-R18 model, representing the best results among all comparison models. More importantly, DSA-DET achieved detection accuracy surpassing all comparison models with only 15.4M parameters while maintaining real-time detection speed of 71.5 FPS. These results fully demonstrate that DSA-DET, through improved network structure design, effectively balances detection accuracy, model complexity, and inference speed, providing a superior solution for intelligent detection of tea diseases.

### 3.6 Recognition model and results visualization

To improve the interpretability of the tea disease detection model, this paper employs the gradient-weighted class activation mapping improvement method Grad-CAM (Selvaraju et al., 2017) to visualize model detection results. Grad-CAM uses gradient information to generate class-discriminative feature response maps, thereby highlighting image regions that contribute most to model predictions. In heatmap visualization results, blue regions represent low model attention areas, green to yellow indicates medium attention, and red to orange regions represent highly attended feature areas. Warmer colors indicate the model considers these regions more important for disease detection.

**Figure 13:**
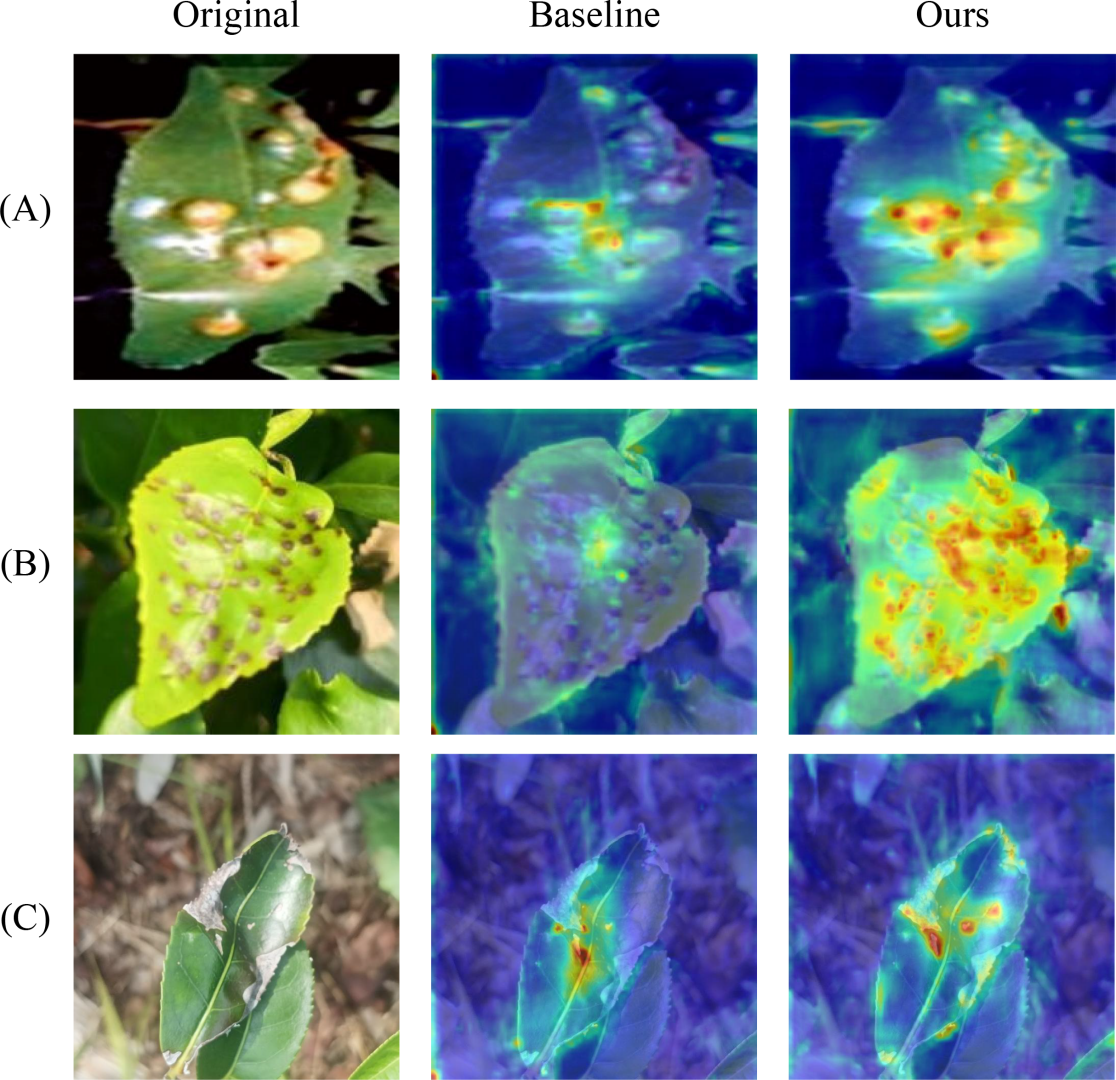
The result of Grad-CAM results. **(A)** Blister Blight. **(B)** Red Rust. **(C)** Brown Blight.

**Figure 14:**
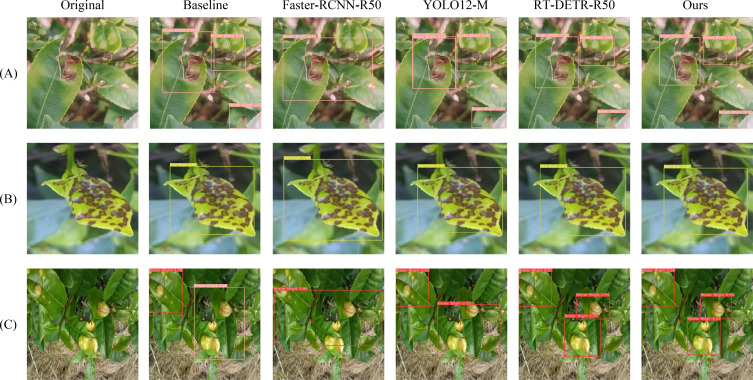
The result of Detection results. **(A)** Brown Blight. **(B)** Red Rust. **(C)** Blister Blight.

**Figure 13** presents the heatmap comparison between the baseline model RT-DETR-R18 and the proposed DSA-DET. The overall heatmap analysis demonstrates that our method exhibits more precise attention focusing capability compared to the baseline model. Attention regions are more concentrated on disease core features, effectively suppressing interference activation from healthy leaf areas and complex backgrounds, while avoiding the scattered attention regions and blurred boundaries commonly observed in the baseline model. Three images were randomly selected for prediction, with overall detection results shown in **Figure 14**. As illustrated in **Figure 14a**, our method performs better in detection completeness, successfully identifying disease regions missed by Faster-RCNN-R50 while achieving superior confidence scores. **Figure 14b** reveals that the proposed DSA-DET achieves higher accuracy than other models and significantly improves boundary localization precision. **Figure 14c** demonstrates that both Faster-RCNNR50 and YOLO12-M exhibit varying degrees of missed detections, with the baseline model also producing false positives.

Comprehensive analysis indicates that the DSA-DET model proposed in this study demonstrates enhanced accuracy and comprehensiveness in detecting tea diseases. The model accurately detects all targets, with detection boxes fitting disease actual contours more closely and overall detection confidence generally higher than the baseline model. It exhibits significant superiority over baseline models in anti-interference capability, detail capture capability, and detection robustness, providing a more reliable technical solution for intelligent diagnosis of tea diseases.

### 3.7 Generalization experiments

The superiority of DSA-DET has been validated through the aforementioned experiments. To further verify the effectiveness of DSA-DET, we conducted a generalization experiment on the Rice Disease Detection Dataset (Xiao et al., 2024), which contains 5,372 training images, 671 validation images, and 672 test images, with three disease categories: Bacterial Leaf Blight, Brown Spot, and Leaf Smut. All experimental parameters were kept consistent with those described above.

As shown in **Table 7**, DSA-DET consistently outperforms all comparison models across all metrics. Specifically, DSA-DET achieves a precision of 95.3%, a recall of 92.8%, an mAP50 of 96.5%, and an F1 score of 94.0%. Compared with the baseline RT-DETR-R18, our model improves precision by 1.8%, recall by 1.5%, mAP50 by 0.7%, and F1 score by 1.6%. Notably, despite the domain shift between rice and tea diseases, DSA-DET maintains superior detection performance, demonstrating generalization across different crop disease detection tasks.

**Table 7:** Results of generalization experiments.

Model	P	R	mAP_50_	F1
*One-stage object detector*				
YOLOV8-M	0.935	0.911	0.957	0.923
YOLOV12-M	0.941	0.918	0.960	0.929
*Two-stage object detector*				
Faster-RCNN-R50	0.925	0.903	0.953	0.914
*DETR-based object detector*				
RT-DETR-R18 (Baseline)	0.935	0.913	0.958	0.924
Ours	0.953	0.928	0.965	0.940

## 4 Discussion

Although the improved DSA-DET model has achieved promising results in the tea disease detection task, certain limitations of this research merit attention. First, individual images in real-world scenarios may encompass multiple disease types, whereas each image in the current dataset provides only a single class label, constraining the training and evaluation of multi-disease scenarios. Second, the current model retains room for improvement in detection stability under extreme lighting conditions and severe occlusion scenarios. Regarding inference efficiency, DSA-DET operates at 71.5 FPS on the AGTea dataset, which is lower than the baseline RT-DETR-R18 yet remains within the real-time detection range. On the Rice Disease Detection Dataset, DSA-DET maintains comparable detection performance, further confirming its generalization capability across different domains. Overall, the proposed method achieves a favorable trade-off between detection accuracy and inference speed for practical deployment. In future investigations, we intend to expand the scale and diversity of the dataset by incorporating additional regions, more tea plant varieties, and composite disease samples to construct a more representative tea disease benchmark dataset. Furthermore, we will explore more advanced algorithmic approaches to address challenges in intelligent agriculture and enhance capabilities for utilizing precision agriculture technologies to meet sustainability goals in agricultural systems.

## 5 Conclusion

In this research, we present DSA-DET, an enhanced tea disease detection framework. Based on the RT-DETR architecture, DSA-DET addresses the challenge of balancing multi-scale feature extraction with real-time inference speed. The framework incorporates three primary enhancements: the DASPMNet module, which utilizes spatial pyramid modeling to detect lesions of various geometries; the HSATE encoder, designed to decrease computational burden through polarized linear attention while maintaining global-local feature characteristics; and the ESCU module, which employs channel shuffling and spatial shifting to improve feature reconstruction compared to the standard RT-DETR-R18. Evaluated on the AGTea dataset, DSA-DET achieved a precision of 94.73%, recall of 89.65%, and mAP_50_ of 93.68%. These results correspond to improvements of 3.56%, 2.96%, and 3.02% over the baseline, respectively, while maintaining a compact size of 15.4M parameters and operating at 71.5 FPS. The performance demonstrates the feasibility of DSA-DET for disease detection in complex field environments, providing a practical solution for precision crop management and smart agriculture.

## Data Availability

The raw data supporting the conclusions of this article will be made available by the authors, without undue reservation.
